# Glioblastoma: Overview and Magnetic Resonance Spectroscopy Analysis for Treatment

**DOI:** 10.7759/cureus.66390

**Published:** 2024-08-07

**Authors:** Waseem Syed, Murat Ibatullin

**Affiliations:** 1 Medical Imaging, Lake Erie College of Osteopathic Medicine, Bradenton, USA

**Keywords:** mr spectroscopy, creatine, astrocytoma, cns tumors, glioblastoma radiology, glioblastoma multiforme, glioblastoma

## Abstract

Glioblastoma multiforme (GBM) is a very aggressive and fast-growing cancer of the brain that has a low life expectancy. Many new cases are diagnosed every year with each having a very poor prognosis. It is therefore of utmost concern to develop cures for such a devastating condition. Magnetic resonance spectroscopy details certain peaks that are of interest. In particular, later-stage astrocytomas exhibit prominent choline and creatine peaks. The creatine peak is known to enhance glioblastoma survival.

## Introduction and background

According to the National Brain Tumor Society, there are more than 100 different types of brain tumors and about one million Americans live with one [1]. The mean survival rate is about 35.7% of those with a malignancy. It is also widely known that brain cancers are one of the most complex cancers to treat because of the very sensitive surrounding structures as well as the relatively impermeably blood-brain barrier (BBB) to various chemotherapy drugs. Of the many different types of brain cancers, glioblastoma multiforme is of interest here.

As explained by Prabhu et al., glioblastoma multiforme (GBM) is a grade IV astrocytoma that is very fast-growing, aggressive, and known to invade local brain tissue [2]. It can either arise from a lower grade astrocytoma or it may arise from anew. GBM is most often seen in the cerebral hemispheres, specifically the frontal and temporal lobes. The life expectancy of this cancer can cause death in less than six months. 

Glioblastoma is the most common malignant brain tumor, which accounts for almost 50% of all CNS tumors. The mean age of diagnosis is 64 years with a 40% chance of survival within the first year of diagnosis and 17% in the subsequent year [1]. As patients advance toward the terminal stage, they eventually lose the ability to eat, sleep, and move. Clinical manifestations of GBM include persistent headaches, diplopia (double vision), vomiting, loss of appetite, changes in mood, changes in cerebration, seizures, and dysarthria. 

Weingart explains that treatment of glioblastoma is done first by surgically resecting the tumor [3]. This is followed by radiation/chemotherapy to improve the chances of survival. However, even with this dual treatment option, the cancers are rarely ever completely gone. 

Diagnosis

**Figure 1:**
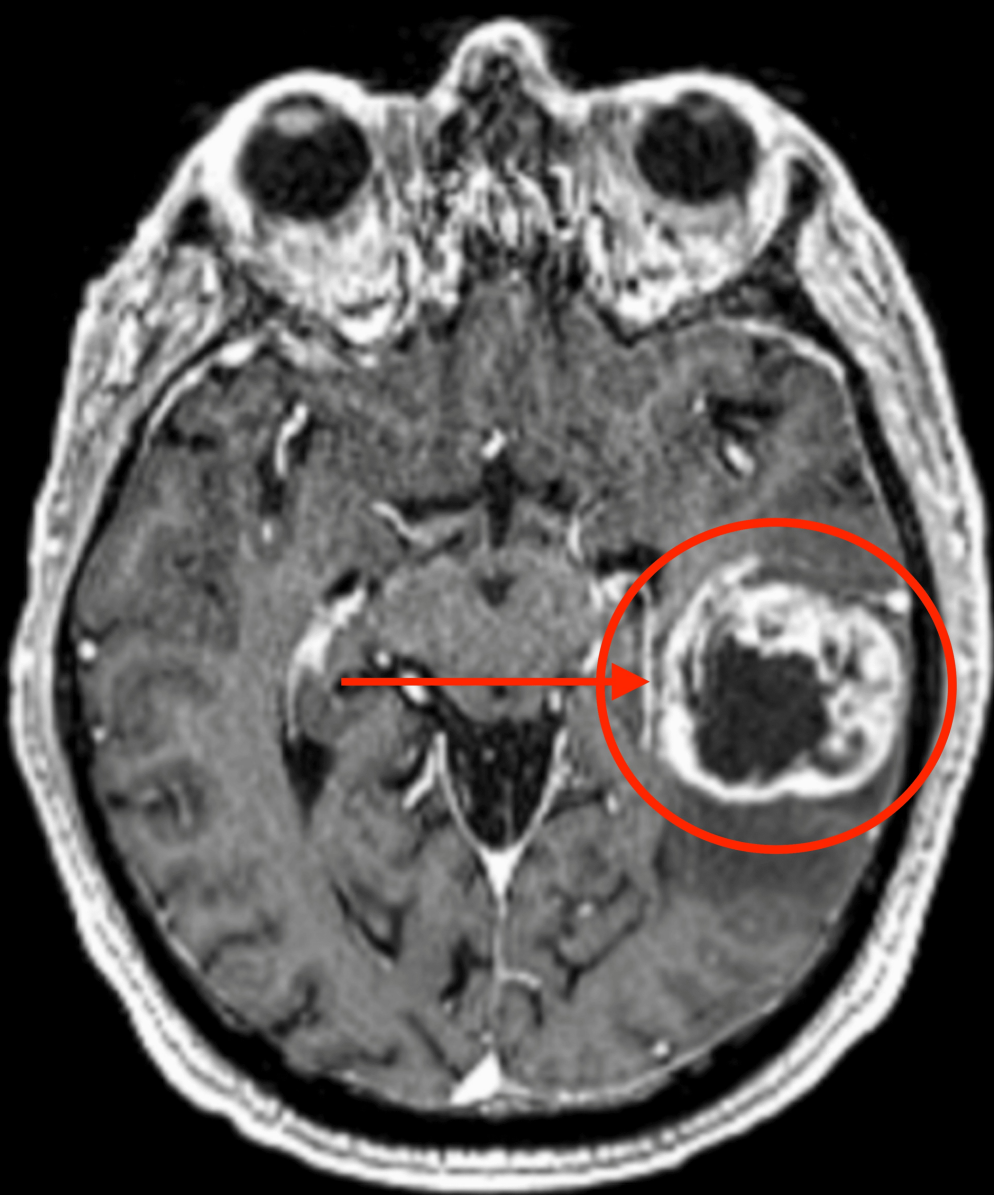
Axial T1-weighted MRIs after IV gadolinium administration The figure depicts glioblastoma and how it looks under a traditional MRI after administering gadolinium contrast using IV. Image Source: American Association of Neurological Surgeons: Glioblastoma multiforme [2]; Used with permission from the American Association of Neurological Surgeons.

Diagnosis must be done with imaging techniques. The most popular studies physicians order are either CT scans or MRIs. A conventional MRI is one of the most important imaging techniques ordered to study astrocytomas. There is usually an IV contrast that is administered, in which higher-grade astrocytomas will take in the contrast and appear bright on the films whereas low-grade tumors do not take up much contrast and will not appear as bright (Figure 1).

**Figure 2:**
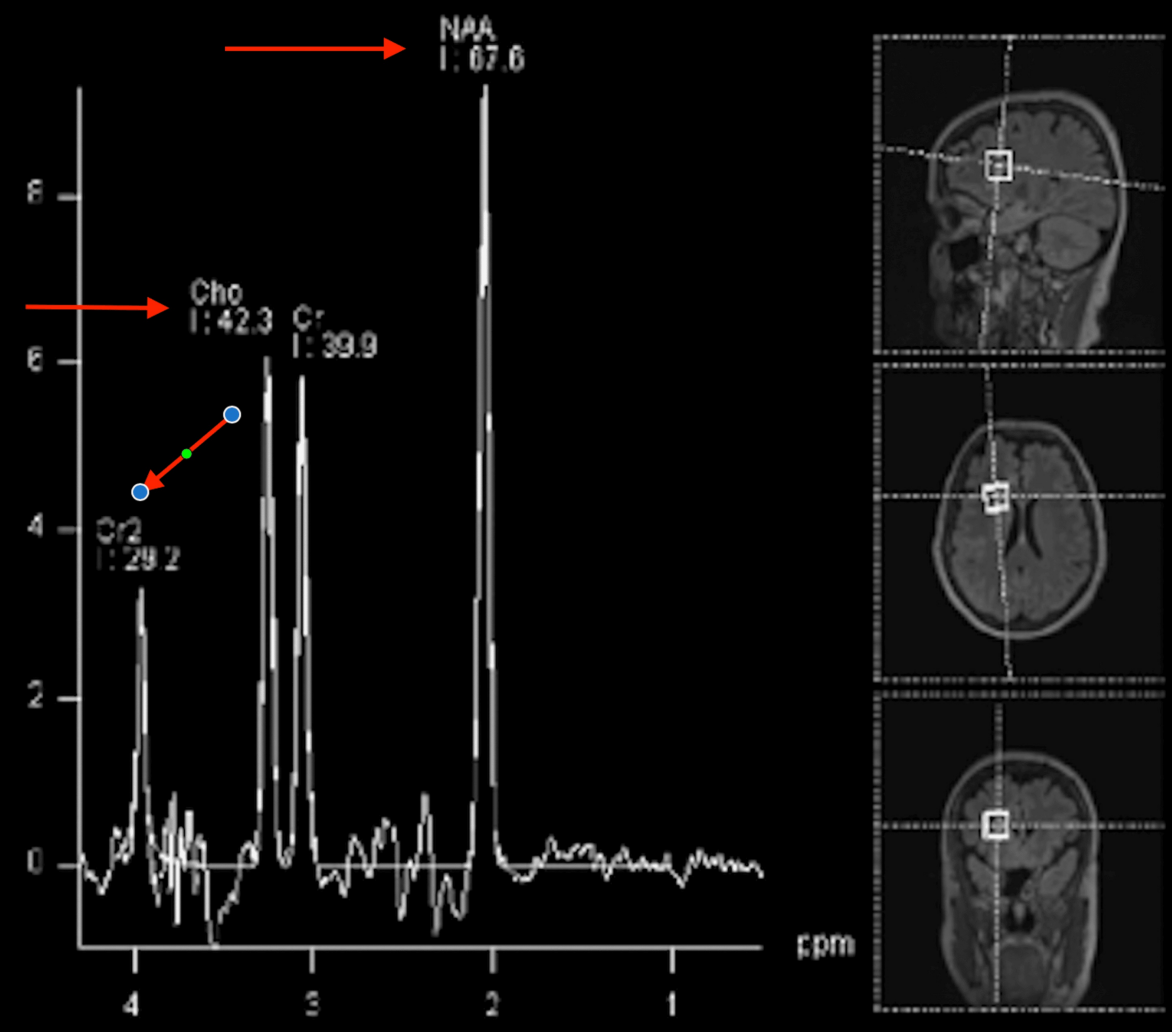
MRS of normal brain (sampled voxels are represented on the right side of the panel). The NAA peak is the most prominent The image depicts a classic view of MRS of a patient with a normal brain. Common peaks seen are NAA, Cho (Choline), and Cr2 (creatine). MRS: magnetic resonance spectroscopy; NAA: N-acetylaspartate Image Source: American Association of Neurological Surgeons: Glioblastoma multiforme [2]; Used with permission from the American Association of Neurological Surgeons.

Magnetic resonance spectroscopy (MRS) provides information on the chemical composition of the tumor in interest. It is done by comparing amounts of N-acetylaspartate (NAA) with choline. In normal brain tissue, the amounts of NAA should be more than choline. However, the inverse may give evidence of a tumor (Figure 2).

**Figure 3:**
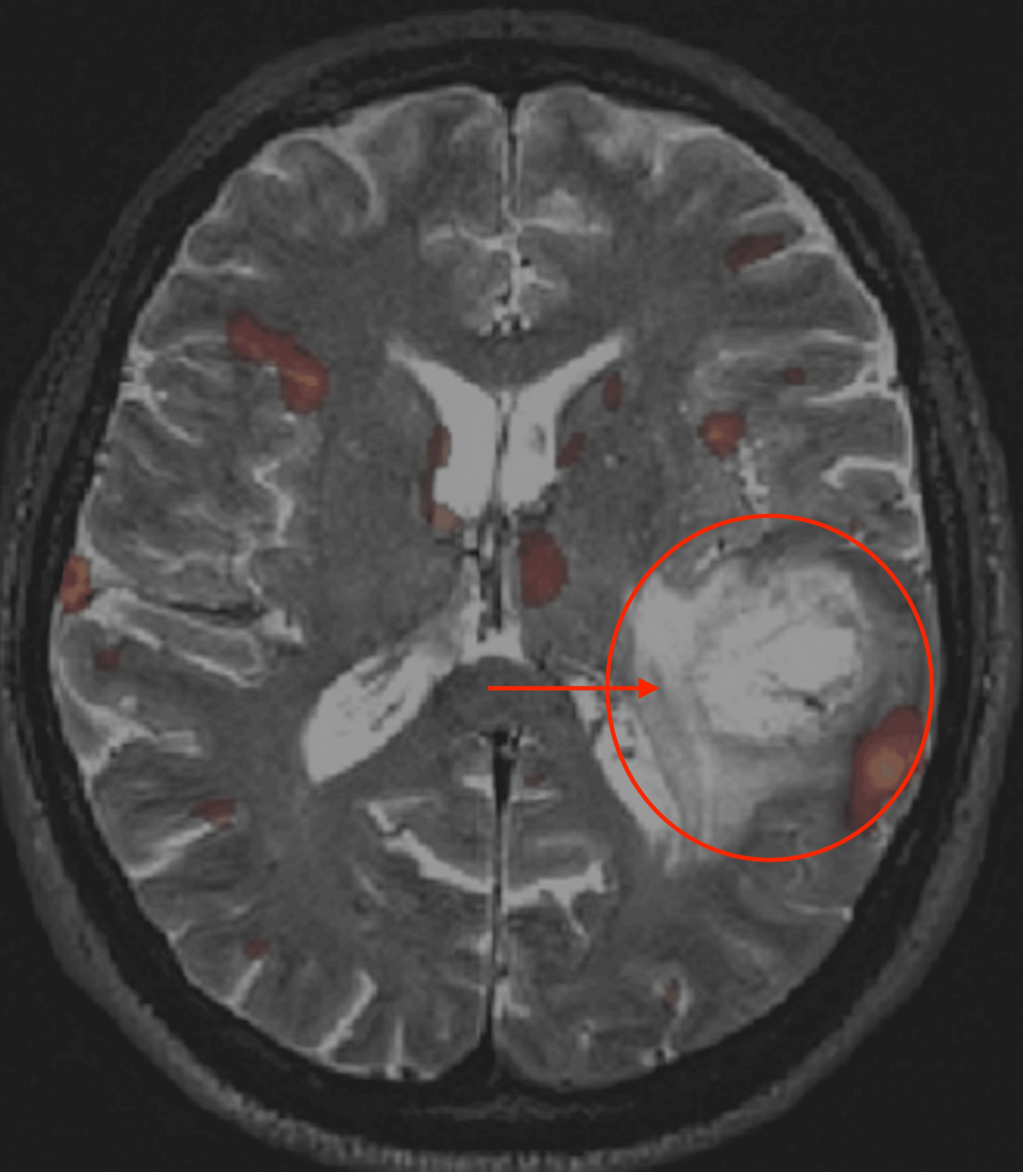
Functional MRI (fMRI) with blood oxygenation level-dependent imaging during object naming The various red spots are areas of the brain that are being used (increased oxygen consumption) during the specific activity, which in this case is naming various objects. Image Source: American Association of Neurological Surgeons: Glioblastoma multiforme [2]; Used with permission from the American Association of Neurological Surgeons.

Functional MRIs are used to see which parts of the brain become active when the patient is asked to do certain tasks. Areas that are deficient in activity when they normally should be animated during a specific task raise suspicion (Figure 3).

The main difference between glioma and glioblastoma is that glioma is more of a broad term for brain tumors that originate from glial cells [4]. Glioblastomas are a type of glioma that specifically involves astrocytes. These are of much higher grades.

The fifth edition of the World Health Organization (WHO) classification of CNS tumors published in 2021 advances the role of molecular diagnostics in the classification of gliomas by emphasizing integrated diagnoses based on histopathology and molecular information and grouping tumors [5]. Astrocytomas are graded from II-IV. Grade II is diffuse astrocytoma. The genes altered are *MYB* and *MYBL1*. It has cytologic atypia, which is a variation in nuclear shape and size along with an abundance of DNA in H&E stains (hyperchromasia). These are also more commonly seen in pediatric patients. Grade III is anaplastic astrocytoma and it shows anaplasia along with increased cellular division. Grade IV is glioblastoma and has a proliferation of vessels along with necrotic tissue. Hemorrhages are a common finding in later stages, although they may be asymptomatic in many different cases. 

The metastasis of glioblastoma occurs in about 0.5% of all glioblastoma cases; however, it should not be taken lightly [6]. The metastatic clusters can deposit in extraneural locations such as leptomeninges and dural venous sinus, as well as extracranial locations such as various organs and lymph nodes. Verification of metastasis can be seen in MRI as it is more sensitive in detecting metastasis. 

**Figure 4:**
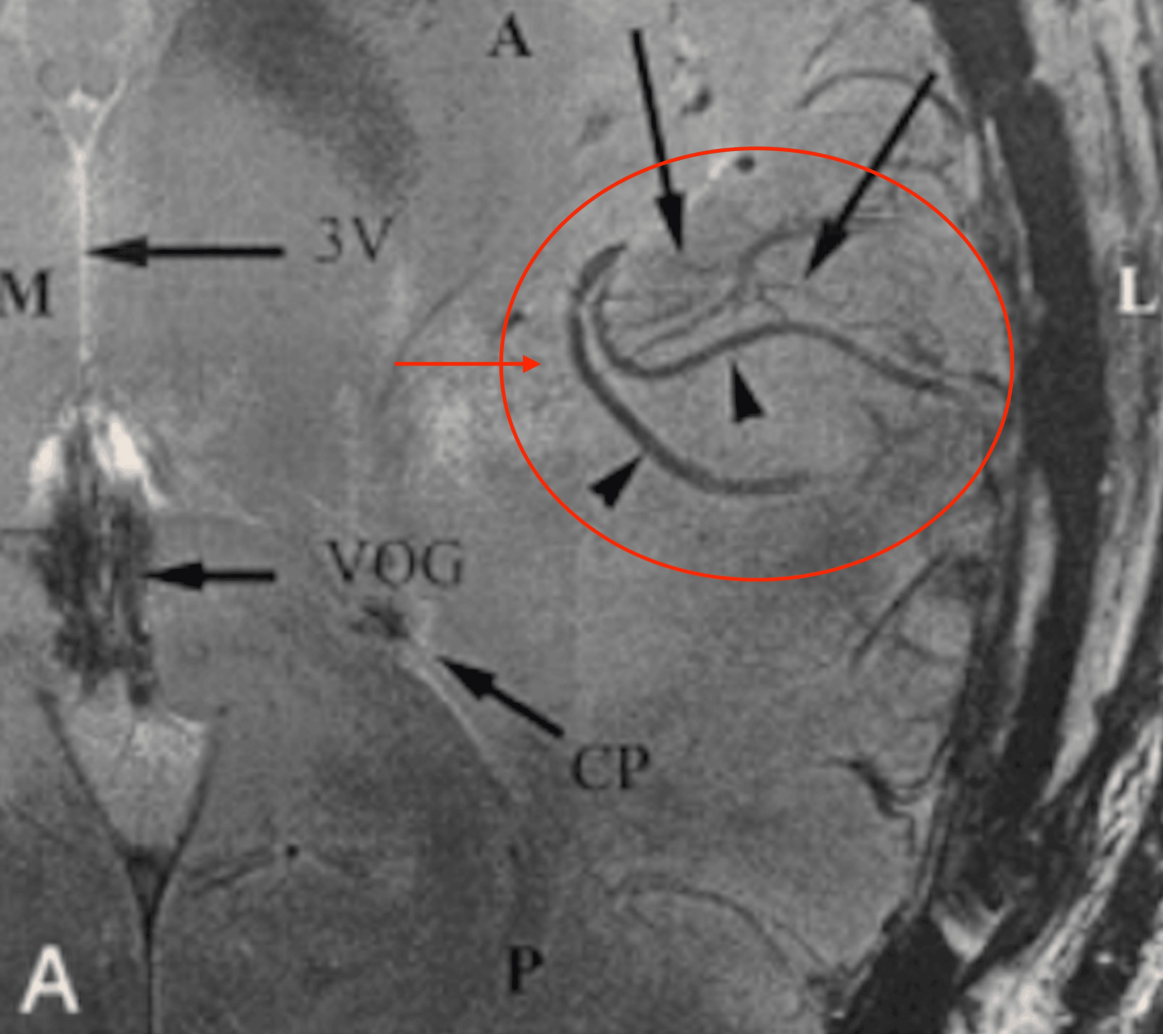
Increased angiogenesis seen in patients with glioblastoma using a standard MRI procedure CP: choroid plexus; VOG: vein of Galen; 3V: third ventricle. Image Source: Christoforidis et al., 2002 [8]; Used with permission from the American Society of Neuroradiology.

**Figure 5:**
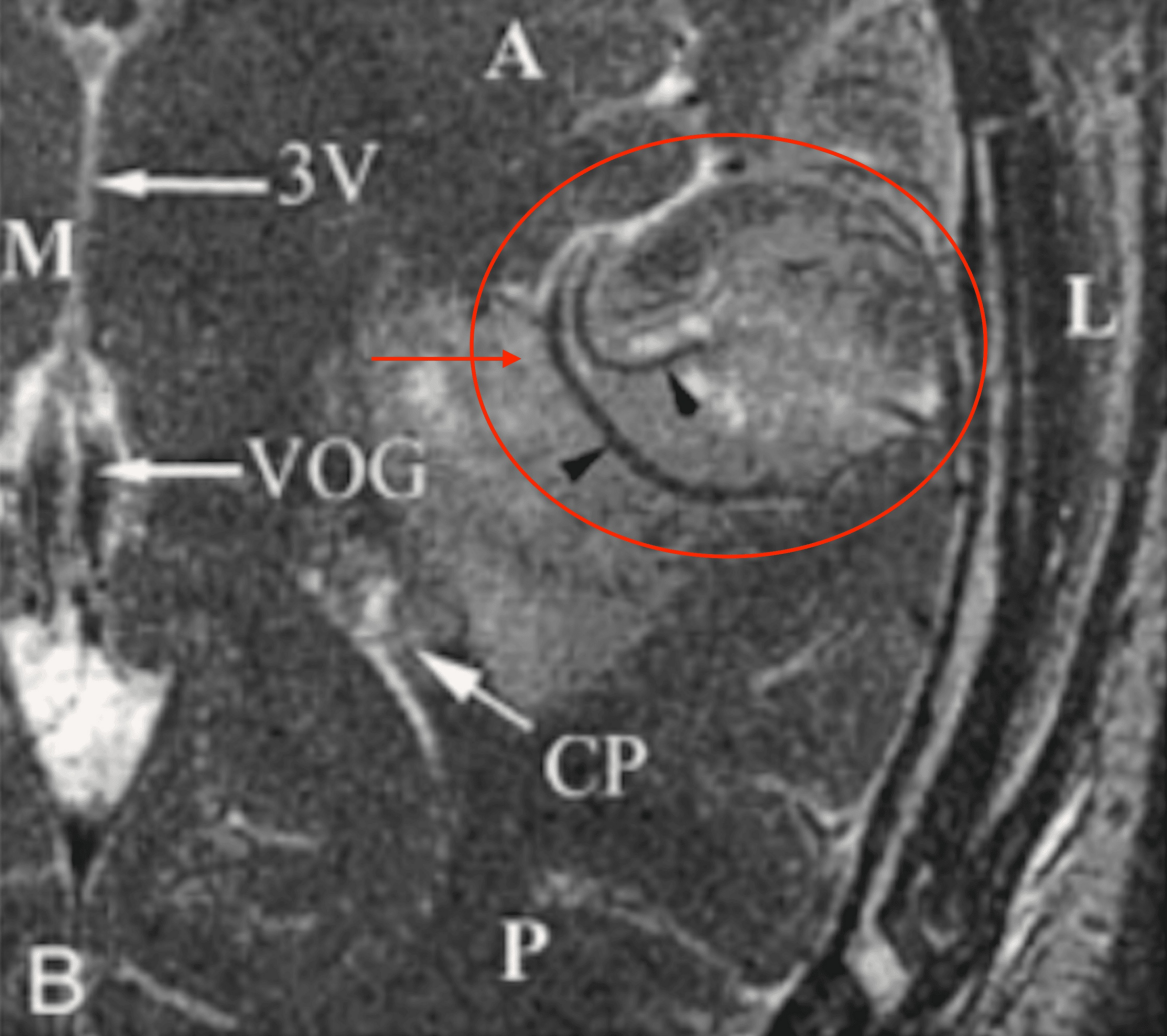
Increased angiogenesis seen in patients with glioblastoma using T2 MRI CP: choroid plexus; VOG: vein of Galen; 3V: third ventricle. Image Source: Christoforidis et al., 2002 [8]; Used with permission from the American Society of Neuroradiology.

**Figure 6:**
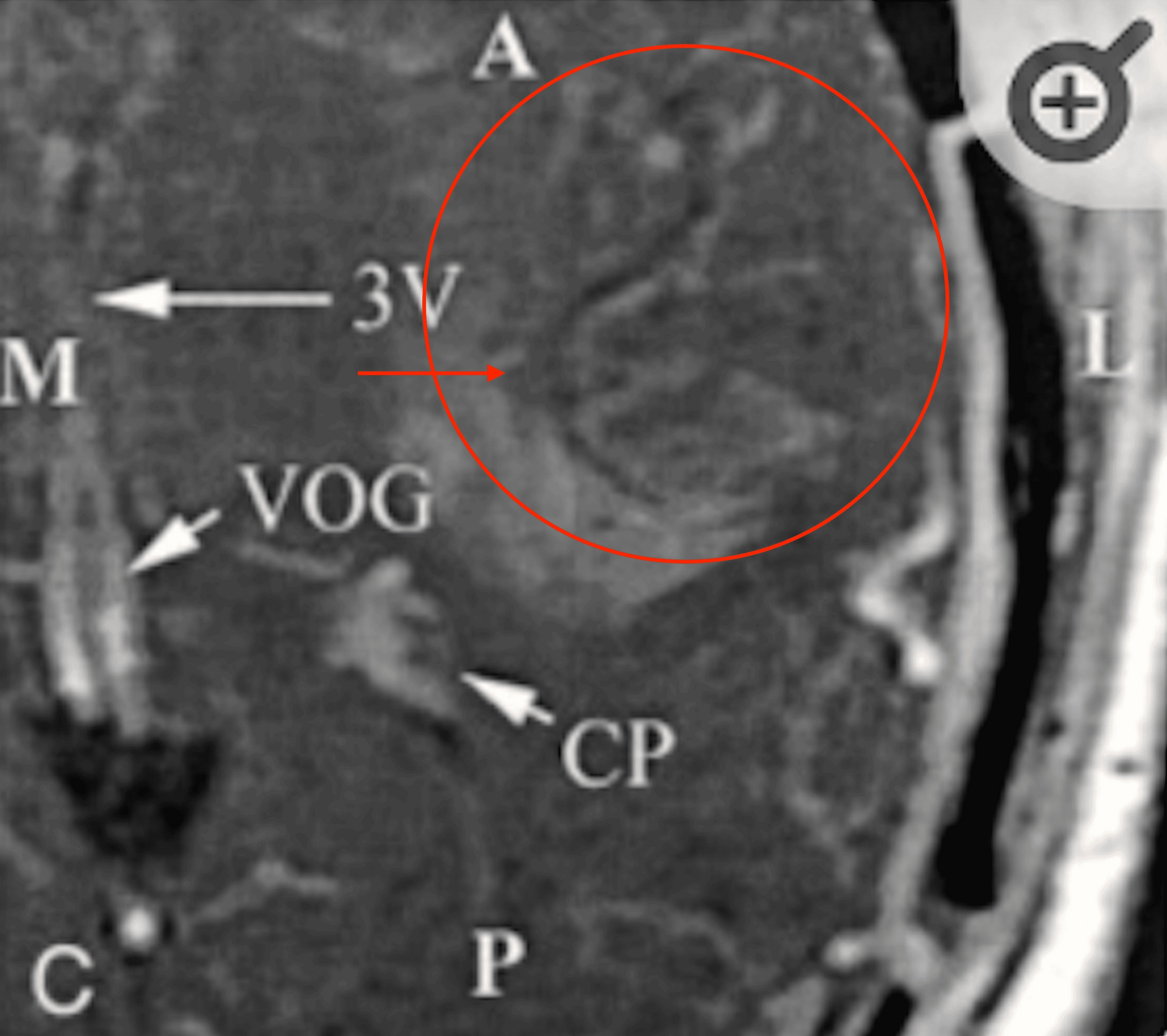
Increased angiogenesis seen in patients with glioblastoma using gadolinium contrast CP: choroid plexus; VOG: vein of Galen; 3V: third ventricle. Image Source: Christoforidis et al., 2002 [8]; Used with permission from the American Society of Neuroradiology.

High vascularization is one of glioblastoma's major features, as is typical of many cancers. The increased blood supply is to feed the growing tumor with increased oxygen and nutrients to fuel its growth. According to Weathers and de Groot, glioblastoma, like all cancers, induces vascular endothelial growth factor (VEGF), which causes increased vascular growth to supply a growing tumor’s needs in a process called angiogenesis [7]. Drugs that inhibit these in target areas can aid in survival rates. Anti-VEGF drugs, such as Avastin and Lucentis, are promising; however, not much progress has been made as far as treatments are concerned. One difficulty of these drugs is being able to cross the BBB to perform its effects. The BBB is extremely selective in only allowing certain substances to cross. Therefore, certain considerations must be implemented when developing treatment options. Figures 4-6 depict the increased vascularization in glioblastoma [8].

## Review

The role of MRS

MRS utilizes the unique distribution of electrons of different compounds and after applying a magnetic field, there is a return signal that is displayed on a graph [9]. There is an X-axis and a Y-axis where the Y-axis measures the chemical shift in units of parts-per-million (ppm). The normal peaks seen are NAA, creatine, and choline. Normal creatine, choline, and NAA peaks form a straight line that, if extended, can create an angle of 45 degrees with the X-axis. This is known as Hunter's angle and can be used to determine if the MRS is normal or not. 

MRS is particularly useful in analyzing primary brain tumors like glioblastoma because of their unique findings. One of them includes a very prominent choline peak. This is because brain tumors are undergoing constant membrane turnover, where choline is present. NAA is derived from neural and axonal tissue. Brain tumors like glioblastoma have a consistent loss of neuronal tissue. Thus, the NAA peak is reduced. 

The common finding in astrocytomas is as the grade increases (from 2 to 4), NAA and creatine peaks decrease while choline, lipids, and lactate peaks increase.

**Figure 7:**
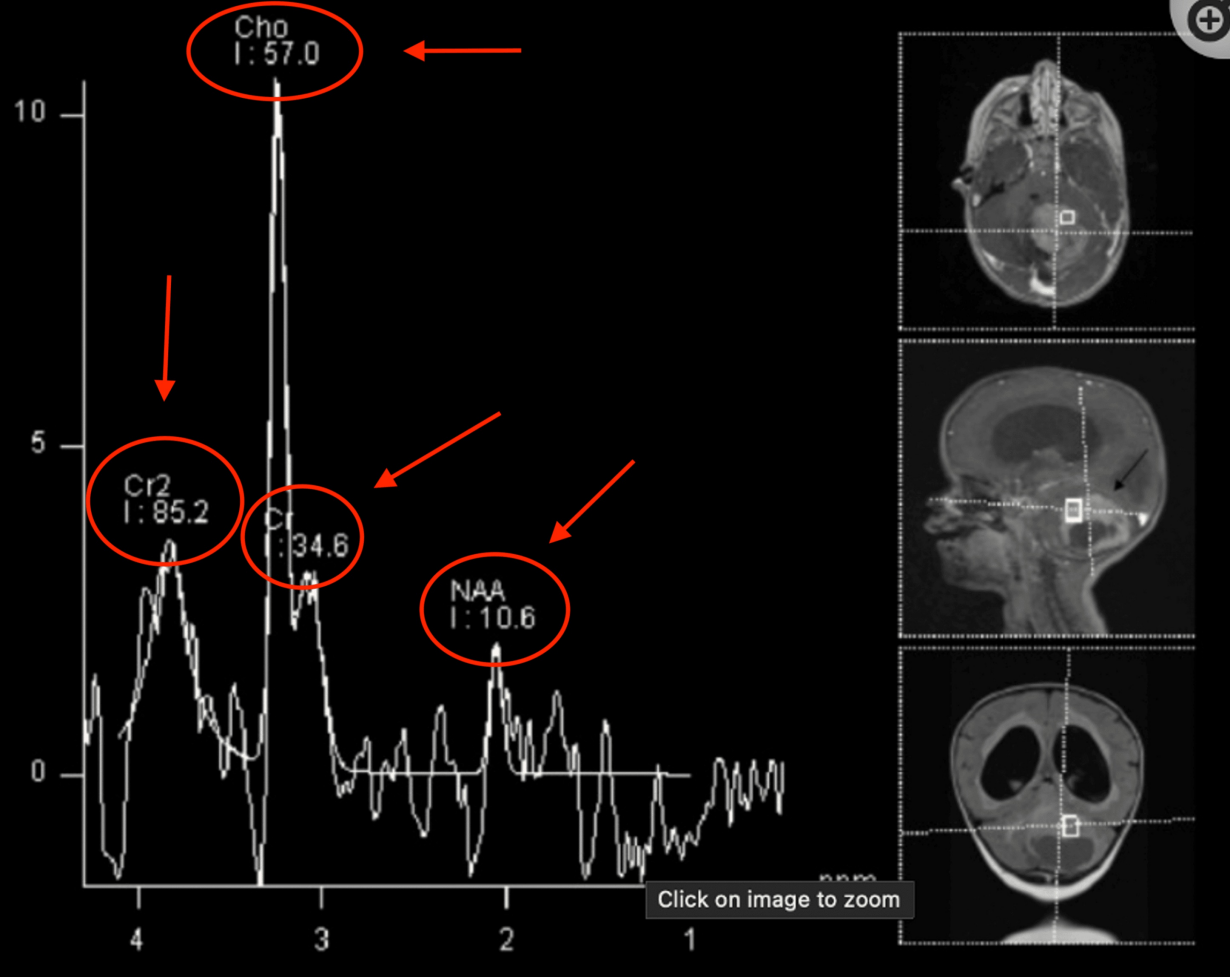
1H-MRS in a patient with pilocytic astrocytoma (black arrow) Significantly increased Cho and decreased NAA and Cr are observed. MRS: magnetic resonance spectroscopy; Cho: choline; NAA: N-acetylaspartate; Cr: creatine Image Source: Galijasevic et al., 2022 [10]; Under Creative Commons Attribution (CC BY) license (https://creativecommons.org/licenses/by/4.0/)

Figure 7 displays the MRS for pilocytic astrocytoma, which is a grade one astrocytoma [10]. Here we can see the prominent choline peak that is consistent with brain tumors due to increased membrane turnover. However, the NAA and creatine peaks are reduced. NAA is reduced due to loss of neuronal tissue, as explained before, and creatine decreases as well. Creatine is known to “feed” the glioblastoma tumor, allowing it to progress to later stages [11].

**Figure 8:**
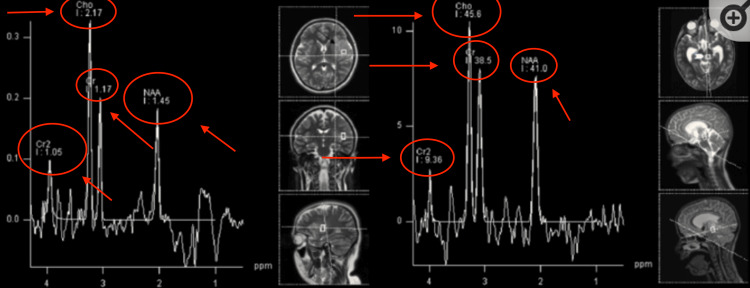
1H-MRS in a patient with astrocytoma WHO grade 3 (left, black arrow) and in a patient with astrocytoma WHO grade 2 (right, white arrowhead) Similar spectra are present in both grade 3 and grade 2 astrocytoma with increased Cho and decreased NAA, with the absence of Cr decrease. MRS: magnetic resonance spectroscopy; Cho: choline; NAA: N-acetylaspartate; Cr: creatine Image Source: Galijasevic et al., 2022 [10]; Under Creative Commons Attribution (CC BY) license (https://creativecommons.org/licenses/by/4.0/)

The image on the right in Figure 8 shows diffuse astrocytoma, which is grade 2. The image on the left shows anaplastic astrocytoma. As the disease progresses from grade 2 to grade 3, there is a reduction of NAA and creatine with the prominent choline peak still present.

**Figure 9:**
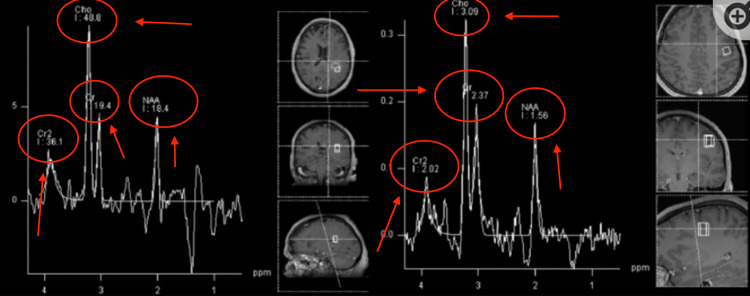
Similar 1H-MRS can be seen in an adult patient with glioblastoma (left, black arrow) and in a patient with astrocytoma WHO grade 2 (right, white arrowhead) with increased NAA and decreased Cr and Cho MRS: magnetic resonance spectroscopy; Cho: choline; NAA: N-acetylaspartate; Cr: creatine Image Source: Galijasevic et al., 2022 [10]; Under Creative Commons Attribution (CC BY) license (https://creativecommons.org/licenses/by/4.0/)

Figure 9 depicts glioblastoma, which is stage 4 astrocytoma. Here we see the same prominent choline peak with decreased creatine with something unique here: increased NAA. 

Effect of creatine

Even though creatine decreases as the stages of astrocytomas progress, it is of interest here because creatine is known to enhance the tumor's survival, induce stem cell proliferation, and ultimately cause progression to later stages. Thus, combating the excessive creation of creatine in GBM can provide for better prognosis. 

**Figure 10:**
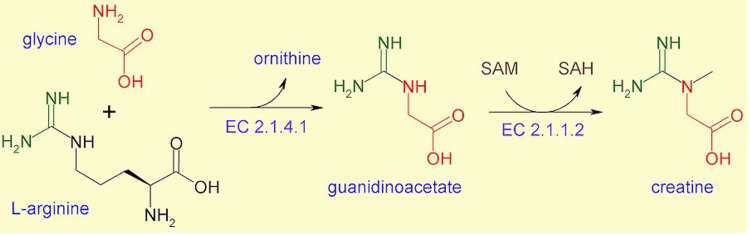
Molecular pathway of the formation of creatine in the body It utilizes the amino acids glycine and L-arginine to form an intermediate, guanidinoacetate. This is later turned into creatine. Image Source: Creatine: Can you finally attain your beach body?, 2022 [12]; Used with permission from the University of Bristol website.

Vasuthan et al. depict how creatine is made in the body (Figure 10) [12]. It is ultimately converted into the final compound from the amino acids glycine and L-arginine. The enzyme EC 2.1.4.1 is the enzyme glycine aminotransferase (AGAT) and the enzyme EC 2.1.1.2 is the enzyme guanidinoacetate N-methyltransferase (GAMT). Both of these enzymes are elevated in patients with glioblastoma. Inhibiting either of these enzymes in the brain can lead to decreased stores of creatine, leading to a potential delay of glioblastoma progression. What is of more interest here is the glycine amidino transferase enzyme because of potential side effects with accumulated guanidinoacetate if EC 2.1.12 (GAMT) is blocked. 

One issue with this mechanism is there are no current inhibitors of either drug. Another issue comes with delivering this product to the brain, as it must pass the highly selective BBB. Nevertheless, it proposes a unique and hopeful solution for higher-grade astrocytomas. 

## Conclusions

Glioblastomas are devastating CNS tumors that have a poor prognosis once a diagnosis is made. Current treatment options include standard surgical resection coupled with radiation/chemotherapy. However, these treatment options most likely delay the inevitable. MRS allows a unique way of analyzing astrocytomas by taking into consideration biochemical changes as the tumor progresses to later stages. Specifically, strong creatine peaks are seen in comparison to normal neural tissue. This creatine enhances the tumor's survival rate and must be tamed in order to prevent further development. Inhibiting enzymes AGAT or GAMT will slow down the creation of creatine, potentially improving the prognosis of those with glioblastoma. Further research is needed to create and implement these enzymes.
